# Surgical Notes from the Army

**Published:** 1862-08

**Authors:** Henry W. Davis

**Affiliations:** Army of Tennessee, Camp Bethel


ARTICLE XXV.

### SURGICAL NOTES FROM THE ARMY.

ARMY OF TENNESSEE,
CAMP BETHEL, *June* 16, 1862.

D. W. STORMONT, M.D.,
*Sec'y Esculapian Society*,

DEAR SIR:—I did not intend that this letter should be prefaced by an apology, as I had many *notes*, taken under a variety of circumstances, varying from the din and smoke of battle, to the cool and quiet of the hospital; from which I *intended* to cull something that I hoped would be of interest. Those *notes*, by *mistake*, are on their way, I trust, to my home in Paris. Why I did not write sooner is due to the fact that I have been waiting for the rebels to lay down their arms and surrender, so that I could leave without a *fight* behind me; and also, from the fact that I hoped to be with you in person at our next meeting, and I may be yet.

The few lines sent will be devoted to generalizations, as I cannot mention special cases; and I will devote them to recording a small part of an experience which has not been small, during the past year. There are questions of importance, which are yet discussed in our Journals. And I shall state my opinions, based upon observation. I cannot go far into the discussion of these things, but will copy the original notes, taken on the field, of two or three cases; "showing the effects of chloroform," and which may assist in settling the point, as to whether it is or is not a "depressing agent."

On Saturday, at Donelson, there was brought to the field-hospital, Alex. G., of Shawneetown; within the previous ten minutes, he had been struck with a missile, which had inflicted a terrible compound injury of the right thigh. The wound through the integuments was apparently incised, in the shape of a Y, and about nine inches in length. The middle and lower thirds of the femur were reduced to fragments, while the flesh within the skin was a pulpy mass. The case determined itself; and amputation was immediately settled upon.

The patient was one of the finest specimens of muscular development I had ever seen; his spirits were buoyant, and he was willing to submit to anything. The question was, "shall we wait until reaction takes place?" as his pulse indicated great physical depression. Nothing else stood in the way of immediate operation; and as the wounded were coming thick and fast, it was determined to proceed. The napkin and sponge holding the chloroform were applied in the usual way; and not more than three inhalations had been taken, before the pulse rose rapidly in volume, and decreased in proportion in velocity. The extent of the injury necessitated a very high operation, and the saw was used within three-fourths of an inch of the trochanter major. So close, indeed, was the resection of the bone made to the articulation, that it was thought, at one time, to remove the head of the femur; but such an operation not being imperative, it was abandoned. The flaps were brought together after a couple of hours; a good stump was obtained; and the man recovered without an untoward symptom; and, to use his own words, in a letter to myself, "there was not a thimbleful of matter from the cut." Here was a case which presented every phase on which a safe opinion might be predicated. The health; the strength; the moral and mental courage; the extent and severity of the injury; the extreme prostration, indicated by the rapid and feeble pulse, and the anxious expression of the countenance; and the prompt and complete reaction on the first administration of the anæsthetic, would lead us safely to the conclusion that chloroform is a stimulent, under certain circumstances. When the vital powers are overcome or held in abeyance by an injury, then it is, the chloroform, like any other agent, calculated to relieve or unloose a chained vitality, becomes indirectly not only stimulating, but supporting in its character. At the close of the operation, and while yet under the influence of the agent, the pulse, in volume and rhythm, was at a fair, healthy standard. I will say here, in connection with this case, that after the removal of the limb from the table, I took from its bed, among the posterior muscles of the thigh, a shell, intact, known as a *twelve pounder*. Its diameter was 4½

or 5 inches, a perfect sphere, without any apparent connection with the incised wound in the skin. The patient sat up and held the edges together, while I inserted the stitches in the flaps.

CASE II.—John E———t, aged 17, was struck with a round ball, one ounce, which grazed the anterior face of the thigh, ranging across and backward, completely severing the left seminal cord, and making an incision in the scrotum, from above downwards, two and a-half inches in length, passing through the perineum obliquely, and lodging against the tuberosity of the ischium, on the right. The nervous depression was very great; pulse ranging from 130 to 140; countenance pale and anxious; disposed to sigh frequently; thirst unquenchable. I was alone; but provided with a field knapsack. I administered chloroform until the pulse rose perceptibly; gave a small dose of brandy and water; and continued the use of the chloroform. While under its influence, I ligated the spermatic artery; removed the left testicle; cut out the ball; closed the wound; and when he was restored to consciousness, neither his pulse or appearance indicated that there was much the matter. He is now with his regiment, the 8th, doing duty.

I can cite a score of similar cases, presenting every degree of severity, but all attended with extreme depression, in which chloroform produced the most salutary effects upon the pulse; and were I brought in connection with a patient, where the use of this agent was demanded, no degree of prostration, however intense, would induce me to withhold or delay its use. I refer, of course, to those cases where the depression is consequent upon recent injury, as it is in this connection that I would discuss its effects.

### AMPUTATIONS, AND OTHER MAJOR OPERATIONS.

This is a heavy text, but I will try to be brief. Experience has taught me that however much good surgery may be taught, there is a good deal of bad, practiced; and a vast amount of unnecessary mutilation. The first question is: What amount of injury will justify an operation? This is, of course, dependant on a thousand outside influences, which must be thrown on the balance for and against. On the field, where an operation is unavoidable, the *sooner the better.* If reaction is slow, *give chloroform;* relieve the pain; remove the effects of the shock; unloose the vital powers, which struggle hopelessly against an overwhelming injury; and then the sooner the system is relieved from the torn and lacerated limb, and a clean cut supplies its place, the better for the patient and the surgeon. *Primary operations are alone successful in saving life,* where it is life or limb, and they must be performed prior to, or during the stage of reaction. They must be performed on the field, before the removal of the patient, or the chances are lessened a thousand fold. There are what are called secondary operations. These are made up of the *removal of a limb,* (after the stage of healthy reaction is passed,) *and the death of the patient.* I will endeavor to make myself understood without being prolix.

When a patient suffers from a compound injury made up of bruised and lacerated flesh and broken bone, without involving the loss of the important vessels, the rule is to *amputate;* the *books* say amputate; professors of surgery, who visit our battle fields with a score of students at their heels, say amputate; and the students at the professor's heels *do* amputate. It matters not to them whether the wound is four hours or four days old, off goes the limb, and out goes the patient; for I challenge denial when I assert that 99 of every 100 of those who were operated on during the *irritative* stage died. This irritative condition commences from 12 to 48 hours after the reception of the injury, and continues from 7 to 20 days, or longer, according to circumstances; and there is no opportunity for the use of the knife during that period, be it long or short. Here, among these cases, conservative surgery finds its field of labor, and the conservative surgeon his element. The knife, for the removal of a limb, is foreign to his thoughts; but every energy is exercised, not only to save life, but to save the important member. He watches his patient closely; sustains his strength; guards against accidents; notes his pulse; and, finally, when the system has become accustomed to the injury, and manifests its regained power and equalized action, by the formation of healthy pus, he asks himself the question, "How little can I venture to throw away?" and acts accordingly. Conservatism, *even in military surgery*, will stand the test; and no sooner is the *itch* for cutting allayed, by an overplus of work, than the most devoted tinkerer with the knife is more disposed to try to save a limb than acquire skill. It may be that I am reviewing only *two* of the *three* sides to the question, and am not judging impartially; but time will, I believe, bear me out fully in all important points.

At Donelson, I worked for four days, in the rear of my division, most of the time dressing the wounds on the spot where the patient fell. During that fight, I dressed 118 wounds, involving many minor operations, and performed 16 capital operations. Not one of the operations performed on the field had an unfavorable termination, while of those where I was *compelled*, as a *dernier resort*, to use the knife, two days after the battle or longer, *scarce one lived.* It is true the latter were few, but they sufficed to prove to me conclusively, that when the first opportunity has been lost, the second rarely or never presents itself: unless connected with some untoward circumstances, rendering extreme measures necessary. Your patient will die if you operate, and if he survives the shock, passes the irritative stage, aggravated by transportation, the conservative surgeon will always have sufficient faith in the skill of nature to help him save a limb, and with it a life.

I will pass from Donelson to the post hospital at Savanna, where our worthy PRESIDENT found me with 1685 wounded soldiers, from the battle field of Shiloh. The surgery at this point was conservative through compulsion. Overwhelmed with the rush of patients, and being compelled to create something out of nothing, it was many days before I could give a thought to other than administrative duties belonging to my position. Among the assistants assigned me were only *two* who had any practical knowledge of surgery. On visiting the hospitals, and taking a general view of all the cases, I could only say, "*Wait*, give them time. For eight or ten days after the battle, I still said wait. Many of the wounds were destructive in their character, and the knife was the only remedy; still I begged them to wait; because not a single case had yet recovered from the shock, or passed the irritative stage which followed. The pulse was rapid and feverish; the face flushed; the wound angry, and discharging a bloody serous fluid; the immediate and adjacent parts to the injury were sensative to the touch, and the whole system controlled by it, and morbidly irritable. I was winning no enviable reputation fast; and yet, knowing this, I begged them to still wait. On the 2nd Monday following the Sunday's fight, four amputations, one of the thigh, one of the leg; two of the arm were performed in a hospital, under the control of two surgeons from the East. On the next day, there were *three* funerals, and on Wednesday *one.* The four cases *died.* The surgeons were alarmed, as they had a right to be; and again I said *wait*, so far as the *rest* are concerned; and wait they did. There were causes operating on these cases, and against them, that I will briefly narrate. Many of the wounded were stricken down on Sunday, and lay on the field for many hours, until the lost ground was retaken. They were piled into ambulances, taken to the boat, and shipped to Savanna. Here they had shelter and food; but long before we could secure the comforts which are indispensable to the *amputating* room, the first *chance* had passed by, and the only hope was to await the second. From the 12th to the 14th day, I commenced work; wine, tonics, and good food had been doing their work, silently but surely; and I trust I may say, without egotism, that many a poor but gallant fellow is living to bless that word, *wait.* It was at this time that Dr. TenBrook dropped in, and put his shoulder to the wheel, with an energy and good-will that lifted *me* out of the slough of despond, into which I was fast drifting, together with a couple of hospital's full of wounded. There were not many operations during his stay; but they were mainly of a character calculated to *illustrate* the subject in hand. There were some amputations of the arm, fore-arm, thigh, and leg. The patients, with but a single exception, had not suffered by the delay, and with that exception, they all recovered from the operation. There was a class of cases of greater interest, from the fact that the injuries primarily were such as to justify amputation; and had they been met at the proper time, with ordinary facilities, even the *conservative* would have been inclined to remove the injured limb. As it was, they slipped through the hands of the surgeon, until they were seen with a view to the performance of an operation, which would save the limb without increasing the risk to the patient's life. With Dr. TenBrook the following operations were performed:—

Lieut. S., gunshot wound, fracturing the *tibia* for 5½ or 6 inches, breaking it into fragments. Twice had a *brace* of *Brigade*-Surgeons met to cut it off, and twice did I enter a protest; the second time, in a very unmistakable manner. The time was not yet for operating, and amputation was *not* the *operation*. After extension was made sufficient to adjust the broken, but not comminuted *fibula*, and allowing time sufficient to allay the local and general irritability, an operation was performed, by which the removal of all the fragments of bone was effected, and the ends of the upper and lower fragments sawed off; under the influence of chloroform he bore the operation well. A simple fracture-box was applied, which allowed complete dressings; after a few weeks, he left for home, with a certain prospect of recovery, with a good, reliable, and useful leg.

J. V. H., a private, was injured by a rifle ball, which shattered the *ulna*, for several inches just below the elbow-joint. The suppuration stage was fully established; his appetite good; and his spirits rather above par. It was one of those cases which might have been operated on a week previous, and which would not have suffered from a few days' delay. In company with Dr. TenBrook, we witnessed the operation, as performed by Dr. ———, the surgeon-general from Wisconsin. The shattered fragments were removed; the ends of the shaft above and below clipped off; and the wound closed. In this case the capsular ligiment was uninjured, and the integrity of the joint undisturbed. The recovery was rapid and complete. There were several cases similar to these, which Dr. TenBrook can give you in detail; and among them all, I do not know of a single case that terminated unfavorably.

During the week following the departure of Dr. T., I was visited by Prof. JOHNSON, of Chicago, Lind University. Time had settled the fate and determined the character of many of the surgical cases in the hospitals, and as the weather was propitious, and all things forward, we put in a week of honest, earnest, and successful labor. Many important operations were performed, carrying the knife and saw through almost the entire range of military surgery. The amputations were few, and bore a small proportion to the aggregate. The question as to the advantages of the circular over the flap operation was fully discussed, and *specimens* of both exhibited; and notwithstanding Prof. JOHNSON is high authority with me, still I will have to see better *recoveries* and neater *stumps* than I have yet, ere I give up the neat, safe, and reliable stump, resulting from the circular operation. There were three resections of the humerus, for injuries to the shoulder-joint; all did well up to the time of my departure, and each would have justified *amputation* on the field. I might go on *ad infinitum*, but I hope, at some future day, not far distant, to present you with a paper on the subject of military surgery, differing I trust from a hastily written letter. I have many *notes*, some I hope of value; but I wish them, even in a new and more carefully prepared dress, to go into the esculapian crucible, before I print them. Some of the ideas deduced from experience will not accord with the surgery of the wars of Napoleon or the Crimea. There is too much surgery practiced, such as it is, and too many mutilations are justified by recognized authority. The surgical history of the army from the North-west is being, or will be, written; and the writer has a splendid theme to urge on his pen. It is to be hoped that the historian will be just and fearless, in the performance of his duty—not his task; and that the Department will never again commit the gross errors which have marked the campaign of 1861-2. More anon.

I am, my dear Dr., with high regard, yours, etc.

HENRY W. DAVIS.

